# The treatment of booking gestational diabetes mellitus (TOBOGM) pilot randomised controlled trial

**DOI:** 10.1186/s12884-018-1809-y

**Published:** 2018-05-10

**Authors:** David Simmons, Jodie Nema, Chloe Parton, Lisa Vizza, Annette Robertson, Rohit Rajagopal, Jane Ussher, Janette Perz

**Affiliations:** 1 School of Medicine, Western Sydney University, Penrith, NSW 2571 Australia; 2 Campbelltown Hospital, Therry Road, Campbelltown, NSW 2560 Australia; 3 Centre for Health Research, Western Sydney University, Penrith, NSW 2571 Australia; 4 School of Medicine, Western Sydney University, Locked Bag 1797, Campbelltown, NSW 2751 Australia

**Keywords:** Gestational diabetes mellitus, Randomised controlled trial, Glucose, Birthweight

## Abstract

**Background:**

We piloted a randomised controlled trial (RCT) comparing pregnancy outcomes among women with booking gestational diabetes (GDM) receiving immediate or deferred treatment.

**Methods:**

Consecutive, consenting women < 20 weeks gestation, with GDM risk factors attending the hospital book-in clinic, completed an oral glucose tolerance test (OGTT). Clinicians were blinded to OGTT results. Women fulfilling World Health Organisation GDM criteria were randomised to either clinic referral /ongoing treatment (Treated Group *n* = 11), or no treatment (No Treatment Group *n* = 10). Women without ‘Booking GDM’ (‘Decoys’ *n* = 58) and those in the No Treatment Group had a repeat OGTT at 24–28 weeks (with GDM treated if diagnosed). Midwives and mothers were asked to complete surveys and attend focus groups before and after the study respectively regarding their experiences and expectations of the study protocol.

**Results:**

Sufficient women completed each step of the RCT. Gestation at OGTT was late at 18 ± 2 weeks with Treated and No Treatment groups largely similar. At 24–28 weeks gestation, GDM was present in 8/9 (89%) in the No Treatment group and 11/56 (20%) Decoys. NICU admission was highest in the Treated group (36% vs 0% *p* = 0.043), largely due to small for gestational age, and Large for Gestational Age babies greatest in the No Treatment group (0% vs 33% *p* = 0.030).

**Conclusion:**

An RCT deferring ‘Booking GDM’ treatment is feasible. Most women with untreated ‘Booking GDM’ in mid 2nd trimester had GDM at 24–28 weeks. Early treatment may have both benefits and harms. A full RCT is needed.

**Trial registration:**

Australia New Zealand Clinical Trials Registry ACTRN12615000974505. Registered 17th May 2015; URL: https://www.anzctr.org.au/Trial/Registration/TrialReview.aspx?id=369100&isReview=true Retrospectively Registered.

**Electronic supplementary material:**

The online version of this article (10.1186/s12884-018-1809-y) contains supplementary material, which is available to authorized users.

## Background

Gestational diabetes mellitus (GDM) increases the risk of obstetric and neonatal complications, with future risks to mother and baby [1–3]. Two randomised controlled trials (RCTs) of GDM management from 24 to 28 weeks gestation, among women without pre-existing diabetes, showed significant reductions in the risk of both macrosomia and pre-eclampsia/Pregnancy Induced Hypertension [4, 5].

It has been recommended that women with possible undiagnosed diabetes are identified early in pregnancy and treated [6, 7]. However, during the screening process, a significant proportion of women are identified as below the biochemical criteria for ‘Diabetes in Pregnancy’, yet fulfilling criteria for GDM at 24–28 weeks gestation. Fasting glucose can be higher at the beginning of pregnancy [8], with one Chinese study reporting that many women with ‘early GDM’ did not have GDM when the oral glucose tolerance test (OGTT) was repeated at 24–28 weeks [8]. This has led to calls not to use the 24–28 week GDM criteria earlier in pregnancy [9]. However, this has left a void, with no recommendations on the next management steps for women with lesser degrees of hyperglycaemia early in pregnancy.

The Treatment of BOoking Gestational diabetes Mellitus (ToBOGM) Study is a randomised controlled trial (RCT) developed to test whether women with ‘hyperglycaemia’ at booking (Booking GDM), should be treated to avoid GDM complications, or whether earlier treatment increases other pregnancy complications. We now report on the findings of the ToBOGM pilot RCT investigating recruitment, proportion with ‘GDM’ at booking, blinding procedures, uptake of heel-prick glucose and outcome measures to facilitate power calculations.

## Methods

The primary aim of this pilot study was to test the protocol for a larger scale RCT of either immediate or deferred treatment for Booking GDM. Clinic midwives at the Campbelltown Hospital Book-in clinic (i.e. first antenatal clinic attendance) assessed the need of all pregnant women for early testing for Diabetes in Pregnancy, based upon GDM Risk factors [10]. Women are referred for a 75 g, 3 point, 2 h oral glucose tolerance test (OGTT) as per local policy. Between July 20th 2015 and April 7th 2016, consecutive pregnant women at 4 to 19 + 6 weeks gestation, with a singleton pregnancy, aged ≥18 years and referred for an OGTT were invited to participate. Exclusions were inability to understand English, or a presence of a major active medical disorder. Consenting women completed questionnaires at baseline, 24–28 and 34–38 weeks and attended a study OGTT appointment. Other data were extracted from the clinical notes.

OGTT results were sent to one investigator (DS). Women with Diabetes In Pregnancy (fasting ≥7.0 and/or 2 h ≥ 11.1 mmol/l) were referred immediately to the clinic for treatment. Women below the 24–28 week GDM [4] criteria (fasting ≥5.1 and/or 1 h ≥ 10.0 mmol/l and/or 2 h ≥ 8.5 mmol/l), were advised that they did not require referral to the clinic (‘decoys’). Such decoys blinded the obstetric team as to whether women involved in ToBOGM were controls or women without ‘hyperglycaemia’. Women fulfilling criteria for GDM were randomised to either referral to the clinic (Treated) or were advised (using identical letters to the ‘decoys’) that they did not require referral to the clinic (No Treatment). The participant, as well as midwifery, obstetric, diabetes clinic, and research staff were kept blinded to all numeric results and only knew if a woman had been referred for GDM treatment. Randomisation to either immediate or deferred treatment was undertaken using an electronic randomiser (SPSS Version 22.0, IBM, USA), stratified as either low risk (fasting 5.1–5.2 mmol/l, 1 h 10–10.5 mmol/l and /or 2 h 8.5–8.9 mmol/l) or high risk (based upon the IADPSG 2 fold excess risk of adverse outcomes at 24–28 weeks gestation [6]: fasting 5.3–6.9 mmol/l, 1 h ≥ 10.6 mmol/l and /or 2 h 9.0–11.0 mmol/l).

All women receiving treatment for GDM received group education, were taught to self-monitor blood glucose and saw a dietitian. Fasting and 2 h post-prandial glucose targets were < 5.3 mmol/l and < 6.8 mmol/l respectively [5]. Where glucose thresholds were exceeded on more than two occasions with no obvious cause, women were offered medication (metformin or insulin treatment). Only women not referred to clinic attended a 24–28 week OGTT, and were referred for treatment if GDM was diagnosed.

All women were asked for their baby to provide a heel prick glucose 1–2 h after birth. Venous umbilical cord blood was drawn into EDTA tubes for assessing a range of metabolites and assessing the adipo-insular axis. Glucose, triglyceride and 3-beta hydroxy butyrate were measured in a single International Organization for Standardization accredited laboratory. Plasma insulin and C-peptide levels were measured using a sandwich chemiluminescence immunoassay (Liaison XL, Diasorin, Saluggia, Italy). Leptin and adiponectin measurements were determined using a radioimmunoassay kit (Merck Millipore, Darmstadt Germany).

### Qualitative assessment

Midwives at Campbelltown Hospital completed a survey prior to the pilot study commencing. An additional one hour focus group was conducted with a group of midwives (*n* = 6) at the conclusion of the pilot study, to examine their experience of taking part in the RCT. A survey was also completed by mothers who were booked into the clinic at Campbelltown Hospital for antenatal care. A group of mothers (*n* = 4) also took part in a focus group at the conclusion of the pilot study. The midwife and the mothers’ surveys both contained demographic questions, closed ended questions about knowledge of GDM and open ended questions about expectations and experiences of the pilot study protocol. In the focus groups, the midwives and mothers were asked about their expectations and experiences of GDM and the study protocol. The focus groups were audio recorded and transcribed verbatim. Focus group transcripts and open ended interviews were analysed using thematic analysis [11]. This involved reading through the qualitative data, developing a coding frame based on commonality of responses across participants, leading to identification of themes that captured expectations and experiences of GDM and involvement in the study. The themes included acceptability, unacceptability, concerns, absence of concerns, and importance of mothers consent.

### Statistics

As a pilot, limited by funding, a target was set to randomise 20 women with GDM at booking, with an expectation, based upon a prior study [12], that 100 women would need to be consented to meet this target. Mean and standard deviations are reported for continuous variables, and number and percentages are reported for categorical variables. Geometric mean is used for non-normally distributed biochemical measurements [13]. All tests are 2 tailed. Analyses were undertaken using SPSS Version 22.0 (IBM, USA) with proportions compared using Chi squared test and continuous variables compared using Analysis of Variance. The study was approved by the South Western Sydney Local Health District Ethics committee (reference 15/LPOOL/14)*.*

## Results

**Fig. 1:**
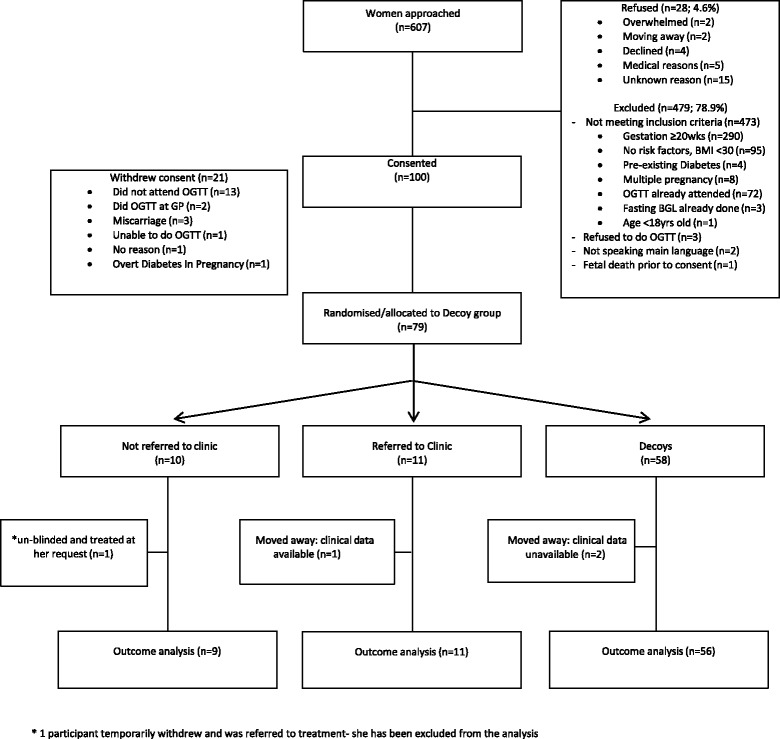
CONSORT diagram: Study uptake at different stages in the ToBOGM Feasibility Randomised Controlled Trial

Figure 1 includes the numbers invited (607), consented (100), and randomised (22). One woman randomised to No Treatment decided to withdraw from the trial and was referred to the GDM clinic and treated. Of the 128 eligible women, 100 (78.1%) consented and 79 (61.7%) entered the study. The randomisation process was adhered to, 88% of babies had the heel prick test and other outcomes were available for 96% of women/babies.

Table 1 compares the Treated and No Treatment groups, and ‘any booking GDM’ with the decoys. Compared with decoys, women with booking GDM had higher baseline BMI, serum insulin, leptin and 3 beta hydroxyl butyrate concentrations and lower adiponectin levels. At baseline, 4/21 (19%) with booking GDM, were diagnosed at the low and 17/21 (81%) at the higher glycaemic stratum; 17/21 (81%) had an elevated fasting glucose level with or without a higher post load glucose. Treated women had a higher systolic blood pressure than No Treatment women at baseline. Among the remaining women randomised to No Treatment, 8/9 (89%) had GDM on the 24–28 week OGTT while 11/56 (19.6%) had developed GDM in the decoy group. There were no differences between the Treated and No Treatment group for gestational age at delivery. Babies of women in the No Treatment group were more likely to have a large for gestational age (LGA) baby (*p* = 0.03), but babies of women in the Treated group were more likely to be admitted to NICU (*p* = 0.043: three small gestational age (SGA), one of whom was < 37 weeks). The only stillbirth was in the No Treatment group with placental abruption (not considered to be due to delayed treatment). Compared with women with booking GDM, decoys had more gestational weight gain (*p* = 0.001) and were less likely to be induced (*p* = 0.001).

**Table 1:** Characteristics of the study participants and their pregnancy outcomes

	Referred to clinic (Rx)	Not referred to clinic (NoRx)	Decoys	Sig GDM vs Decoy^b^	Sig referred to clinic vs not referred to clinic at Booking^c^
N Baseline/ Outcomes	11/11	10/9 ^a^	58/56		
Age	29(5)	30(7)	28(5)	0.181	0.746
Non Europid	4 (36.4%)	5(50%)	30(51.7%)	0.486	0.528
University degree	2 (18.2%)	1(10%)	11(19%)	0.630	0.593
Family History of Diabetes	4 (36.4%)	3(30%)	25(43.1%)	0.435	0.757
Smoker	0 (0%)	2(20%)	9(15.5%)	0.497	0.119
Gestation on entry (weeks)	17.0(2.1)	15.7(3.1)	15.3(2.5)	0.114	0.277
Gestation at OGTT (weeks)	18.5(1.2)	17.5(1.8)	17.5(2.0)	0.307	0.173
Systolic BP(mmHg)	111(11)	101(8)	106(12)	0.857	*0.029*
Diastolic BP (mmHg)	64(7)	63(9)	64(9)	0.837	0.693
Height (cm)	164(6)	164(8)	163(8)	0.790	0.948
Weight (kg)	87.2(23.7)	89.9(26.4)	77.8(20.9)	0.057	0.807
BMI (kg/m^2^)	32.3 (7.8)	33(7.0)	28.9(6.6)	*0.034*	0.824
Fasting glucose (mmol/L)	5.1(0.4)	5.2(0.3)	4.6(0.3)	*< 0.001*	0.464
1 h glucose (mmol/L)	8.0(1.7) (*n* = 10)	8.4(1.6)	6.7(1.4)	*< 0.001*	0.602
2 h glucose (mmol/L)	7.0(1.9) (*n* = 10)	6.8(1.7)	5.6(1.2)	*0.001*	0.790
Maternal Fasting Insulin (pmol/L)	118.2	122.4 (*n* = 9)	59.8 (*n* = 53)	*< 0.001*	0.910
Maternal Fasting Cpeptide (pmol/L)	426.5	686.9 (*n* = 9)	412.1 (*n* = 53)	0.190	0.379
Maternal Fasting Adiponectin(μg/mL)	7.2(2.4)	9.5(2.9) (*n* = 9)	12.1(7.5) (*n* = 53)	*0.028*	0.071
Maternal Fasting Triglyceride(mmol/L)	1.8(0.6)	1.9(0.5) (*n* = 9)	1.7(0.7) (*n* = 51)	0.370	0.748
Maternal Fasting Leptin(ng/mL)	57.6(30.0)	69.4(25.0) (*n* = 9)	46.1(25.4) (*n* = 53)	*0.016*	0.360
Maternal Fasting 3 BHB(μmol/L)	96.9	98.3 (*n* = 9)	64.0 (*n* = 53)	*0.002*	0.950
GDM at 24–28/40	–	8/9 ^a^(89%)	11/56(19.6%)		
Insulin and/or metformin	4/11 (36%)	4/10(40%)	3/11(27.3%)	0.443	0.864
Gestation at birth (weeks)	38.7(1.4)	39.2(0.6)	38.5(2.2)	0.440	0.326
Gestational weight gain	5.3(3.7)	8.1(2.5)	10.4(5.3)	*0.001*	0.074
Pre-eclampsia/pregnancy induced hypertension	3(27%)	0(0%)	6(10.7%)	0.664	0.089
Male baby	6(55%)	6(67%)	22(39%)	0.160	0.582
Induction of labour	7(64%)	3(33%)	9(16%)	*0.001*	0.178
Emergency CS Elective CS	4(36%) 1(9%)	1(11%) 2(22%)	7(13%) 14(25%)	0.236 0.313	0.194 0.413
Neonatal Intensive Care Unit admission	4(36%)	0(0%)	8(14.3%)	0.608	*0.043*
Heelprick Blood glucose (mmol/l)	3.0(0.7) (n = 9)	3.3(0.8) (*n* = 8)	3.1(1.0) (*n* = 46)	0.995	0.515
Neonatal Glucose % ≤ 2.2 mmol/l	1/9 (11%)	1/8(13%)	6/46(13%)	0.834	0.929
Weight of baby (g)	3055(758)	3552(743)	3339(682)	0.873	0.159
Average Centile	46(39)	57(35)	56(34)	0.801	0.531
<10th Centile	3(27%)	0(0%)	5(8.9%)	0.493	0.089
>90th Centile	0	3(33%)	14(25%)	0.583	*0.030*

^a^One woman withdrew and was referred for treatment: she consented to data being collected and is included in the GDM vs Decoy comparison but not the Referred vs Not Referred comparison

^b^Sig GDM vs Decoy = significance of difference between all women with and without GDM at booking

^c^Sig referred to clinic vs not referred to clinic at Booking = significance of difference between women with GDM at booking who were or were not referred to clinic

Mean APGAR 1 min and 5 min =9(1); Not referred: one shoulder dystocia

Statistically significant comparisons shown in italics

The characteristics of midwives and women participating in the survey and focus groups are shown in Additional file 1: Table S1 and Additional file 2: Table S2. Of the women, one was a participant in the pilot study, one was currently pregnant but did not have GDM, one had recently had a baby and did not have GDM, and one had GDM in a previous pregnancy. All were familiar with the OGTT testing in pregnancy. Tables 2 and 3 describe the qualitative responses of midwives and antenatal women respectively from the open ended survey questions and focus groups. Overall, the midwives’ and the mothers responded positively to the study procedures. Respondents gave feedback on the importance of informed consent throughout all stages of the study. Most women reported preferring face-to-face initial contact. There were some concerns around the ethics of delaying treatment. The heelprick glucose was widely accepted. In the focus group, the midwives talked about the need to have a researcher dedicated to the study in the clinic, so that the study did not impact on their workload.

**Table 2:** Midwives’ perspectives on key study procedures – qualitative responses

What are your thoughts on the study specifically approaching women in the booking clinic to participate, who have been identified as at risk for GDM?	*Acceptable* • Recruitment strategy is ‘good’, ‘good idea’ or ‘fantastic idea’ (S: 7) *No concerns* • No concerns regarding the ethics of approaching this group (F: 3) • Neutral or no concerns recruitment (S: 6) *Concerns* • Consent is an important issue, women should be aware of what their participation would involve e.g. blinding of results, potential withholding of treatment (F: 3, S: 1) • Do not agree with the deliberate recruitment of an at risk population (S: 1)
What do you think about delaying treatment, if needed, to 24 to 28 weeks?	*Acceptable* • Treatment timing is acceptable/appropriate (S: 8) • Acceptable, as standard procedures used to be based on 28 weeks to detect GDM (S: 1, F: 1) • More information/research needed on the outcomes of delayed treatment (S: 2) *Concerns* • Concern about the legal and ethical issues of providing adequate ‘duty of care’ to patients whose results were withheld due to research participation (F: 2) • Concern that researchers could make a mistake in identifying at-risk patients and delaying treatment (S: 2) • No issue unless GDM is detected earlier, then treatment should not be delayed (S: 3) *Not acceptable* • Not acceptable, testing and treatment should be made earlier to prevent risk to the mother and foetus e.g. could be too late to treat, to educate mother, prevent complications (S: 4) • Detection and treatment should occur earlier only if risk factors are present (S: 2) • Treatment delay could prevent mothers from experiencing unnecessary anxiety about their pregnancy (F:3) • Unsure (S: 1)
How do you feel about the heel prick test for glucose at one hour old?	*Acceptable* • Midwives were not concerned as it was not part of their workload (F: 2) • Baby’s best interests and health the main priority (S: 1) • Test is needed and therefore there should be no concerns (S: 5) • Not necessary as already part of standard care (S: 4) • Test okay if managed professionally (S: 1) • Test should occur on the basis it will assist in health research (S: 1) *Importance of mothers’ consent* • Mothers’ consent/refusal must be respected (S: 7) • Mothers’ should be educated and given opportunity to consent, prior to birth (S: 4) • Issues with consent can only be dealt with if there is ‘medical indication’ that test must be done, and a subsequent “refusal of treatment” is put in clinical notes (S: 1) • Concern that test may not be conducted due to mothers’ viewing it as unnecessary despite previously consenting (F: 2) *Concerns* • Test may not be undertaken due to staff being overworked/too busy (S: 1) • Unnecessary for babies who do not have a diabetic mother (F: 1)

*S* Survey responses, *F* Focus group responses

**Table 3:** Mothers’ perspectives on key study procedures – qualitative responses

What are your thoughts on the study specifically approaching women in the booking clinic to participate, who have been identified as at risk for GDM?	• Women described the recruitment process positively and had no objection to being approached (S: 4) • Clinic recruitment was considered a good research strategy (S: 2) • Women described that the recruitment process was a positive as it raised awareness of GDM in regards to education on diet, complications and prevention (S: 2) • Women liked that recruitment was conducted by a person, as it aided the establishment of rapport and understanding of the study (F:2, S: 1) • Flyers or pre-clinic booking would help give mothers time before being approached in clinic to make a decision (S: 2) • Mothers preferred being approached face-to-face as opposed to via a flyer or email (F: 2) • Face-to-face increases chances of participation (F: 1)
What do you think about delaying treatment, if needed, to 24 to 28 weeks?	*Good or okay to delay* • Participants described delayed treatment as a good idea (S: 6, F:1) • Participant indicated that if the early GDM test was not necessary it could save Medicare resources (S: 1) • Mothers preferred to be only tested at 28 weeks, as it is standard practice in many places and in the past (F: 1) • Mother approved of the delay, as she thought her own early testing and treatment was invasive and unnecessary (F: 1) • Participants approved, as GTT was so unpleasant it should only be conducted once (F: 1) *Not sure* • Not sure (S: 5) *Should not delay* • GDM should not be delayed if it can be treated early (S: 1. F: 1) • Mother would still want to check at booking (S: 1) • Delaying treatment would be detrimental to mother and child as there is no need to change the current approach to treatment at booking (S: 1) • Participant expressed that if detected some treatment should occur, even if that is just a modified diet (S: 1, F: 1) • Treatment should not be delayed (S: 1, F: 1)
How do you feel about the heel prick test for glucose at one hour old?	*Good or okay* • Good, no problems with test (S: 10, F: 3) • Mothers describe having no objection based on the notion that test will aid in checking health of baby/identification of diabetes (S: 2, F:1) • Only if necessary (S: 7) • No objection as babies have lots of tests when born anyway (S: 2, F:1) • Mothers described that babies forget pain quickly (F: 2) *Not approve or ambivalent* • Not comfortable with test (S: 3) • Mother described being uncomfortable if the test disturbed the baby (S: 1) • Opposed ‘I refuse to see that’ (F:1) • This test should have second consent obtained (F: 2) • Mothers suggested that less blood be collected (F:1)

*S* Survey responses, *F* Focus group responses

## Discussion

We have shown that our study protocol was feasible, requiring few changes. Randomisation and each step of the protocol were implemented with no significant problems. Both the mothers interviewed and the midwives were generally positive about the procedures, although there was some concern over the delay in treatment for women allocated to the No Treatment condition. In spite of this, most eligible women consented to the RCT and were able to enter the study.

Our overall capture rate for the heel prick was over 80%, higher than the 71.25% in HAPO [14]. Those not captured included three premature births that occurred at a neighbouring hospital, a stillbirth, and the remainder not collected due to participants being discharged early and lost to follow-up. We hope to improve the heel-prick capture rate through providing more information to midwives and by providing participants with leaflets/bags with the study logo so that midwives are aware of their involvement in the study and the importance of capturing the heel-prick.

Although small, our study is the first to show that early GDM treatment could be associated with a play off between a reduced LGA rate but an increased NICU admission rate (largely associated with SGA for those treated from booking). SGA/fetal undernutrition can be a consequence of overtreatment [15], or insufficient gestational weight gain [16], with putative long term consequences [17]. Hypertension and smoking (and potentially metformin treatment [18]) can also contribute to reduced fetal growth and in the main trial, it will be important to assess any interaction between these characteristics, fetal gender and outcomes from early GDM treatment. Our study reminds us that we do not know the optimal glycaemic targets early in pregnancy and that we should be cautious. Our study is also the first to challenge the finding that 33–66% of women early in pregnancy might not have GDM on repeat fasting glucose testing [8]. As 89% of untreated women in our study had GDM at both 18 weeks and 24–28 weeks gestation, there is a case for using the 24–28 week criteria from the middle of the second trimester.

Our study has the strength of obstetrician blinding and the use of decoys, to avoid confounding of the NICU admission and LSCS rates. The use of quantitative and qualitative measures is also a strength. The study only tests the use of the IADPSG/WHO criteria [6, 7] and not other criteria for GDM. Weaknesses include the higher baseline systolic blood pressure and the small sample size. The latter prevents the identification of the impact of any specific management issues such as the use of metformin or treatment compliance. The late clinic booking of women was a major impediment to recruitment and we have tested the benefits/harms with a mid-second, not a first trimester GDM diagnosis. Most (48%) of the exclusions were due to this late booking, which was due to local clinic processes (now rectified). For the main study, we have negotiated with the antenatal clinic to expedite the booking of women with risk factors for GDM, to avoid delays and hopefully recruit before 12–14 weeks gestation. Such earlier recruitment is more likely to directly answer the question about the benefits/risks of treatment in the first trimester raised by both Zhu et al. [8] and Sweeting et al. [19]. The latter showed that the group with the poorest pregnancy outcome were diagnosed before 12 weeks. Women diagnosed with GDM between 13 and 23 weeks had outcomes more similar to those diagnosed > 24 weeks. The study would have recruited the target number of women quicker if an early OGTT had been requested universally. However, the study followed the national guideline to use risk factor screening to assess who should proceed to an early OGTT.

## Conclusion

In conclusion, we have completed the first RCT of whether GDM, defined by 24–28 week criteria, should be treated at booking. Our surveys and interviews of mothers and midwives, along with the successful completion of the study highlights its feasibility. Although a small study, the findings suggest that there may be a play off between reducing macrosomia and increasing fetal undernutrition, a finding warranting a larger RCT. A full trial is urgently required.

## Additional files

Additional file 1: **Table S1.** Characteristics of Survey and focus group participants: Midwives (DOCX 13 kb)

Additional file 2: **Table S2.** Characteristics of Survey and focus group participants: antenatal women (DOCX 14 kb)

## Abbreviations

EDTA: Ethylenediaminetetraacetic acid
GDM: Gestational diabetes mellitus
IADPGS/WHO: International association of diabetes and pregnancy study group/world health organisation
LGA: Large gestational age
NICU: Neonatal intensive care unit
OGTT: Oral glucose tolerance test
RCT: Randomised controlled trial
SGA: Small gestational age
ToBOGM: Treatment of booking gestational diabetes mellitus
