# Supplementary material for: The treatment of booking gestational diabetes mellitus (TOBOGM) pilot randomised controlled trial
Source: BMC Pregnancy Childbirth. 2018 May 10;18:151. doi: 10.1186/s12884-018-1809-y (PMC5946423; doi:10.1186/s12884-018-1809-y)
Supplement: Supplementary file 1 — Table S1. Characteristics of Survey and focus group participants: Midwives (DOCX 13 kb) [file 12884_2018_1809_MOESM1_ESM.docx]

Additional file 1: Table S1

Characteristics of Survey and focus group participants: Midwives

|  | Midwife Survey | Midwife Focus Group |
| --- | --- | --- |
| n | 26 | 6 |
| Age <25 | 1 | 0 |
| 25-34 | 6 | 2 |
| 35-44 | 6 | 1 |
| 45-54 | 9 | 3 |
| 55+ | 3 | 0 |
| Non response | 1 | 0 |
|  |  |  |
| Current position |  |  |
| Certified/registered/ caseload midwife | 18 | 4 |
| New grad/student | 4 | 0 |
| Specialist midwife/ nurse/unit manager | 4 | 2 |
| Nursing training | 25 | 6 |
| Full time | 12 | 2 |
|  |  |  |
| Seen women with gestational diabetes in clinic | 22 | 6 |
| Seen women with gestational diabetes in Labour | 24 | Question not asked |
| Adverse clinical outcome with a woman with GDM | 16 | Question not asked |
