# Supplementary material for: The treatment of booking gestational diabetes mellitus (TOBOGM) pilot randomised controlled trial
Source: BMC Pregnancy Childbirth. 2018 May 10;18:151. doi: 10.1186/s12884-018-1809-y (PMC5946423; doi:10.1186/s12884-018-1809-y)
Supplement: Supplementary file 2 — Table S2. Characteristics of Survey and focus group participants: antenatal women (DOCX 14 kb) [file 12884_2018_1809_MOESM2_ESM.docx]

Additional file 2: Table S2

Characteristics of Survey and focus group participants: antenatal women

|  | Antenatal women Survey | Women in Focus Group |
| --- | --- | --- |
| n | 30 | 4 |
| Age <20 years | 1 | 0 |
| 20-29 | 12 | 1 |
| 30-39 | 16 | 2 |
| 40+ | 1 | 1 |
| Ethnic group |  |  |
| Anglo Australian | 13 | 3 |
| Aboriginal Australian | 2 | 0 |
| Asian/ Sth American/ Pacific Islands/Arabic | 11 | 1 |
| European | 2 | 0 |
| No response | 2 | 0 |
| Number of weeks pregnant |  |  |
| <10 | 2 | 0 |
| 11-14 | 1 | 0 |
| 15-18 | 7 | 0 |
| 19-20 | 15 | 0 |
| >20 | 4 | 1 |
| No response | 1 | 3 |
| Number of past pregnancies |  |  |
| 0 | 6 | 0 |
| 1 | 11 | 1 |
| 2 | 4 | 3 |
| 3 | 4 | 0 |
| 4 | 1 | 0 |
| 5+ | 3 | 0 |
| No response | 1 | 0 |
| In paid work | 16 | Question not asked |
| Any family with diabetes? | 15 | 2 |
| Past GDM | 4 | 2 |
| Overweight | 14 | Question not asked |
| Big baby in past | 5 | Question not asked |
